# Supplementary material for: Growth history leaves a geometric trace in puzzle cells
Source: EMBO Rep. 2026 Apr 15;27(10):2559–80. doi: 10.1038/s44319-026-00755-y (PMC13219716; doi:10.1038/s44319-026-00755-y)
Supplement: Supplementary file 1 — Table EV1 [file 44319_2026_755_MOESM1_ESM.pdf]

Table EV1. Species used for the analyses clasiffied by clade.

early diverging angiosperms	eudicots	ferns	gymnosperms
Annona montana	Acanthus hungaricus	Actiniopteris semiflabellata	Araucaria
Chloranthus	Acanthus spinosus	Adiantum hispidulum	Araucaria bidwillii
Drimys winteri	Acer platanoides	Angiopteris	Bowenia serrulata
Illicium anisatum	Achillea millefolium	Angiopteris evecta	Chamaecyparis thyoides
Laurus nobilis	Aconitum carmichaelii	Asplenium adiantoides	Cycas revoluta
Magnolia denudata	Adromischus	Asplenium lancifolium	Encephalartos transvenosus
Magnolia lotungensis	Alliaria petiolata	Asplenium vieillardii	Ginkgo biloba
Peperomia	Alternathea dentata	Bolbitis	Gnetum montanum
	Amaranthus caudatus	Bolbitis lonchophora	Lepidozamia peroffskyana
	Amaranthus hybridus	Ceratopteris thalictroides	Microcycas calocoma
	Anchusa officinalis	Cyrtomium falcatum	Picea abies
	Anemone canadensis	Davallia fejeensis	Picea glauca
	Anthemis arvensis	Davallia solida	Podocarpus macrophyllus
	Ardisia crispa	Davallia subsolida	Taxodium distichum
	Astragalus falcatus	Dicksonia antarctica	Taxus canadensis
	Bellis perennis	Diplazium plantaginifolium	Tsuga canadensis
	Bergenia purpurascens	Doodia media	Widdringtonia nodiflora
	Berkheya purpurea	Elaphoglossum	Zamia furfuracea
	Berkheya radula	Goniopteris	Zamia pumila
	Beta trigyna	Helminthostachys zeylanica	Zamia skinneri
	Beta vulgaris	Lecanopteris sinuosa	
	Borago officinalis	Lygodium japonicum	
	Bougainvillea	Lygodium microphyllum	
	Brugmansia suaveolens	Marattia attenuata	
	Bupleurum fruticosum	Microsorum pteropus	
	Bursera schlechtendalii	Nephrolepis	
	Callicarpa japonica	Niphidium crassifolium	
	Calotropis gigantea	Osmunda banksiifolia	
	Calystegia sepium	Pellaea paradoxa	
	Campanula fenestrellata	Pellaea viridis	
	Campanula poscharskyana	Phanerophlebia	
	Capsella bursa-pastoris	Phlebodium aureum	

Capsicum annuum
Cardamine flexuosa
Carica papaya
Cassia spectabilis
Cataranthus roseus
Catha edulis
Catharanthus roseus
Ceiba pentandra
Centaurea cineraria
Centaurea cyanus
Centranthus ruber
Cephalaria flava
Ceratostigma plumbaginoides
Ceratostigma willmottianum
Cerinthe minor
Ceropegia sandersonii
Chenopodium bonus-henricus
Choisya ternata
Cinnamomum camphora
Cissus quadrangularis
Cissus tiliacea
Citrus limon
Clerodendrum thomsoniae
Cordia nitida
Crassula
Crataegus monogyna
Cuphea ignea
Dahlia pinnata
Dillenia indica
Dodonaea viscosa
Dorycnium rectum

Platycerium andinum
Platycerium elephantotis
Polypodium
Pteris ensiformis
Rumohra adiantiformis
Sphaeropteris cooperi
Stenochlaena palustris
Tectaria
Tectaria pseudosinuata
Todea barbara

Epiphyllum

Ercilla spicata

Eryngium agavifolium

Eryngium bourgatii

Erysimum scoparium

Erythrina standleyana

Euphorbia flanaganii

Euphorbia mellifera

Euphorbia pulcherrima

Fagraea berteroana

Forsythia suspensa

Fraxinus excelsior

Fuchsia magellanica

Fuchsia triphylla

Fuschia Mrs Popple

Galanthus nivalis

Galium odoratum

Galium rubioides

Geranium pusillum

Geum triflorum

Globularia punctata

Globularia trichosantha

Grevillea flexuosa

Guaiacum officinale

Haloragis erecta

Hedera nepalensis

Heimia myrtifolia

Helleborus orientalis

Helminthotheca echioides

Hiptage benghalensis

Homalocladium platycladum

Hypericum patulum

Hypericum perforatum

Ilex aquifolium

Ilex paraguariensis

Impatiens balsamina

Impatiens repens

Ipomea tricolor

Jacquemontia tamnifolia

Jasione heldreichii

Jasminum fruticans

Jasminum humile

Justicia guttata

Lactuca sativa

Lamium orvala

Lamium purpureum

Leucanthemum vulgare

Linaria vulgaris

Lithocarpus henryi

Lonicera quinquelocularis

Macleania insignis

Malva sylvestris

Medicago sativa

Mentha x piperita

Mespilus germanica

Mimosa pudica

Montinia caryophyllacea

Murraya koenigii

Mussaenda glabra

Myrrhis odorata

Nemophila menziesii

Nicotiana tabacum

Nyctanthes arbor-tritis

Oenothera glazioviana

Oenothera stricta

Othonna capensis

Oxalis latifolia

Oxalis triangularis

Oxalis valdiviensis

Oxyria digyna

Paeonia tenuifolia

Papaver rhoeas

Passiflora

Passiflora edulis

Pastinaca sativa

Pelargonium carnosum

Penstemon

Persicaria polystachya

Persicaria weyrichii

Physalis origanifolia

Plantago afra

Poliothyrsis sinensis

Polygonum affine

Populus tremula x tremuloides

Potentilla reptans

Prunus spinosa

Psephellus simplicicaulis

Psidium guajava

Rauvolfia verticillata

Rhus potaninii

Ribes sanguineum

Rosa x damascena

Rubia tinctorum

Rumex acetosella

Rumex scutatus

Sambucus nigra

Saxifraga canaliculata

Saxifraga hostii

Scabiosa olgae

Scorzonera hispanica

Scrophularia canina

Scrophularia heterophylla

Securigera varia

Senna didymobotrya

Silene latifolia

Sisymbrium austriacum

Solanum nigrum

Stachys macrantha

Strobilanthes wallichii

Succisella inflexa

Symphytum caucasicum

Symphytum grandiflorum

Symphytum orientale

Syringa

Syringa vulgaris

Tabernaemontana divaricata

Taraxacum officinale

Tecoma stans

Thunbergia mysorensis

Tilia cordata

Torrenia asiatica

Trifolium pannonicum

Ulex europaeus

Valeriana phu

Verbena bonariensis

Veronica gentianoides

Veronica missurica

Veronica persica

Veronica petraea

Veronica rakaiensis

Viola odorata

**Miocene species**	**monocots**
Araliaceae-Pseudopanax	Acorus gramineus
Atherospermatacea-Laurelia otagoensis	Agapanthus praecox
Elaeocarpaceae-Cunoniaceae	Agave sisalana
Lauraceae-Cryptocarya maarensis	Albuca bracteata
Lauraceae-Cryptocarya sp	Aloe
Lauraceae-Cryptocarya taieriensis	Alpinia zerumbet
Lauraceae-Endiandra	Alstroemeria aurea
Lauraceae-Litsea calicarioides	Amaryllis belladonna
Meliaceae-Dysoxylum	Amorphophallus titanum
Myrtaceae	Avena strigosa
Primulaceae-Myrsine	Calibanus hookeri
Ripogonaceae-Ripogonum	Carex morrowii
Unknown-Morphotype O	Carludovica palmata
	Clivia miniata
	Crocus sativus
	Crocus vernus
	Cymbidium
	Danae racemosa
	Danthonia californica
	Dioscorea bulbifera
	Dioscorea mexicana
	Eichhornia crassipes
	Eriachne
	Eustrephus latifolius
	Freycinetia
	Haworthia retusa
	Hemerocallis fulva
	Hordeum vulgare
	Hydrocleys nymphoides
	Iris
	Iris albicans
	Iris japonica

Kniphofia caulescens

Leucojum aestivum

Oryza sativa

Pandanus tectorius

Rohdea japonica

Ruscus aculeatus

Ruscus hypoglossum

Sabal minor

Setaria italica

Smilax bona-nox

Sorghum bicolor

Sternbergia lutea

Stichoneuron caudatum

Tradescantia virginiana

Tricyrtis hirta

Triraphis

Yucca gloriosa

Zea mays
