# Supplementary material for: Growth history leaves a geometric trace in puzzle cells
Source: EMBO Rep. 2026 Apr 15;27(10):2559–80. doi: 10.1038/s44319-026-00755-y (PMC13219716; doi:10.1038/s44319-026-00755-y)
Supplement: Supplementary file 2 — Appendix [file 44319_2026_755_MOESM2_ESM.pdf]

Appendix

Growth history leaves a geometric trace in puzzle cells

Trozzi N, Lane B, Perruchoud A, Clark F, Hoermayer L, Meraviglia A, Reichgelt T, Roeder AHK, Kwiatkowska D, Runions A, Smith RS and Majda M.

Table of contents

Appendix Fig. S1. Growth rates used in simulations shown in Figures 1 and 2. 2

Appendix Fig. S2. Regional variation in pavement cell lobeyness across Arabidopsis leaf. 3

Appendix Fig. S3. Landmarks and cellular tracking in maize growth. 4

Appendix Fig. S4. Correlations between the min-axis and lobeyness in various mutants and with LEC in drug-treated plants 6

Appendix Fig. S5. Lobeyness of pavement cells is negatively correlated with cell division activity. 7

Appendix Fig. S6. Developmental variation in leaf pavement cell shapes. 8

Appendix Fig. S7. Variation in epidermal pavement cell morphology on the organ surface. 9

Appendix Fig. S8. The lobeyness of pavement cells varies between adaxial and abaxial leaf surfaces. 10

Appendix Fig. S9. Organ-specific differences in pavement cell shape between leaves and floral structures. 11

Appendix Fig. S10. Correlation patterns between min-axis and lobeyness in pavement cells within individual species 12

Appendix Fig. S11. Visual examples of pavement cell contours demonstrating high, low, and negative correlations between lobeyness and min-axis. 14

Appendix Fig. S12. The analysis of LEC across different species suggests a correlation between cell size and lobeyness 15

Appendix Fig. S13. Distribution of α values across species. 17

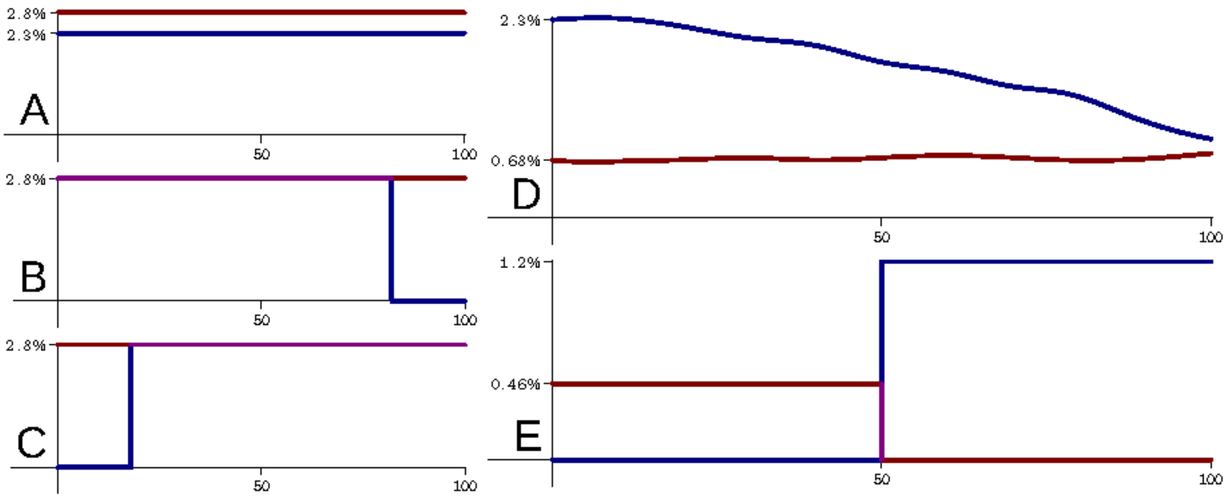

Appendix Fig. S1. Growth rates used in simulations shown in Figures 1 and 2. All simulations ran for 100 time steps. Graphs show relative expansion per time step in X (blue) and Y (red) directions. (**A–C**) Total growth was 10-fold in the X direction and 16-fold in the Y direction. (**A**) Uniform anisotropic growth (Figure 1D, G). Growth rates are constant over time, with growth in Y slightly higher than in X. (**B**) Isotropic growth followed by anisotropic growth (Figure 1E, H). Growth in Y is constant over the entire growth period. Growth in X matches it until the final width is reached, at which point growth in X ceases. (**C**) Anisotropic growth followed by isotropic growth (Figure 1F, I). The same as **B**, but the X growth period comes at the end of the simulation rather than the beginning. (**D–E**) Total growth was 6-fold in the X direction and 2-fold in the Y direction. (**D**) Growth rates drawn from measurements of maize (Figure 2P). The growth rate in the Y dimension remains stable. The growth rate in X starts high and then decreases over time. (**E**) Maize template with very different growth rates (Figure 2Q). All growth in Y occurs in the first half of the simulation, while all growth in X occurs in the second half.

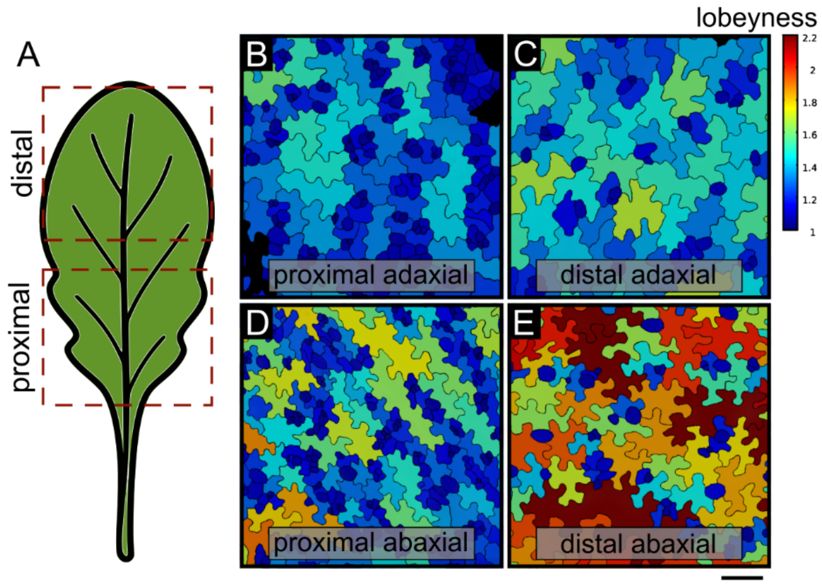

Appendix Fig. S2. Regional variation in pavement cell lobeyness across Arabidopsis leaf. (**A**) Schematic of a mature Arabidopsis leaf showing red dashed boxes demarcating the proximal and distal sampling regions on both the adaxial (upper) and abaxial (lower) surfaces. (**B–E**) Lobeyness heatmaps of segmented pavement cells in each indicated region: (**B**) proximal adaxial, (**C**) distal adaxial, (**D**) proximal abaxial, (**E**) distal abaxial. In each panel, individual cells are color-coded by lobeyness (warmer colors = higher lobeyness), illustrating that lobing is more pronounced in distal versus proximal regions and more elevated on the abaxial surface compared to the adaxial surface. Scale bar, 50 µm (**B-E**).

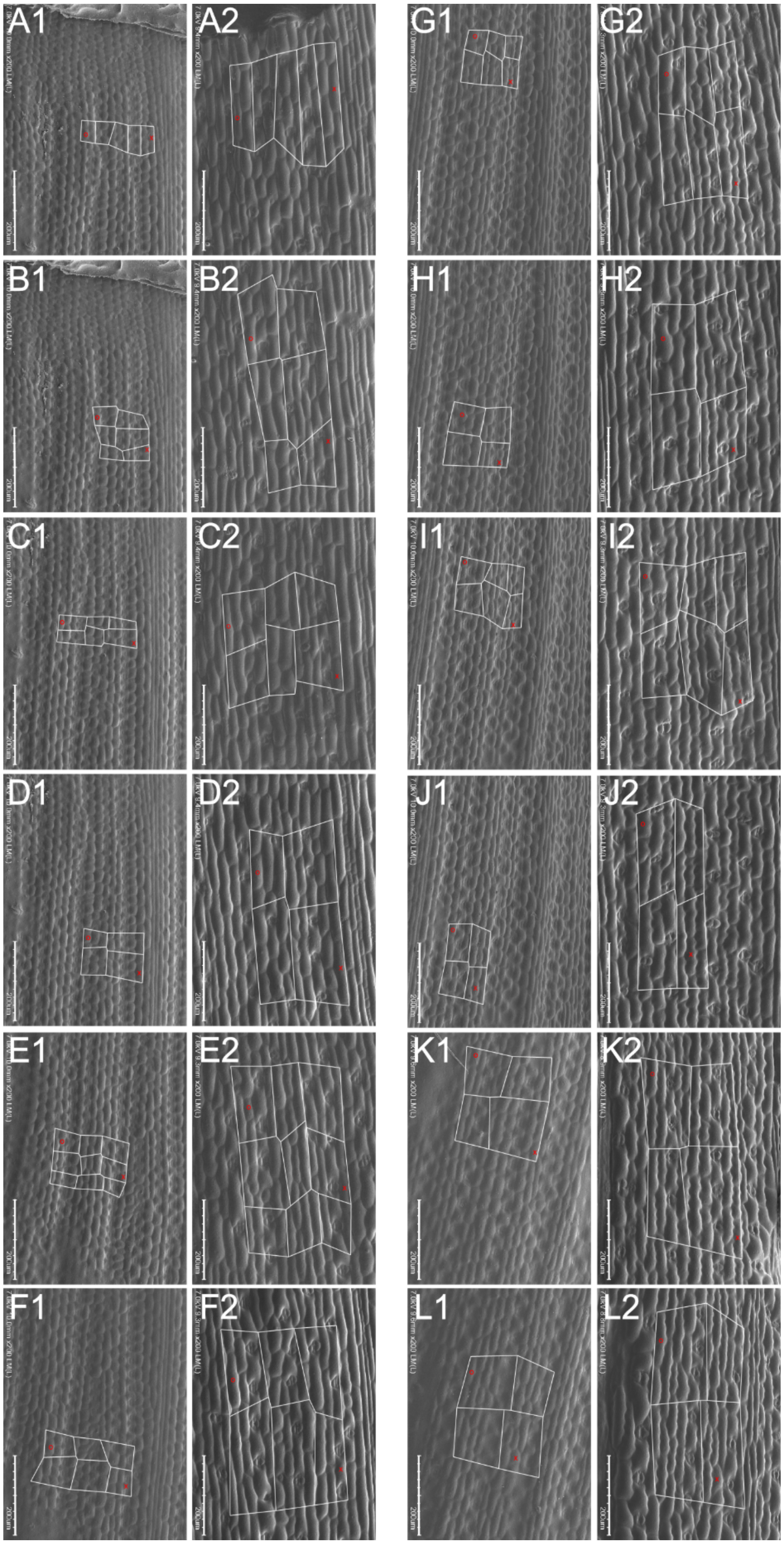

Appendix Fig. S3. Landmarks and cellular tracking in maize growth. (A1-L2) Electron micrographs, as in Figure 2, with landmarks used to calculate growth shown. Specific cells are

marked with red O or X in each paired set (*e.g.*, A1 and A2) to track individual cells at 0 h and 24 h. Scale bars, 200 μm.

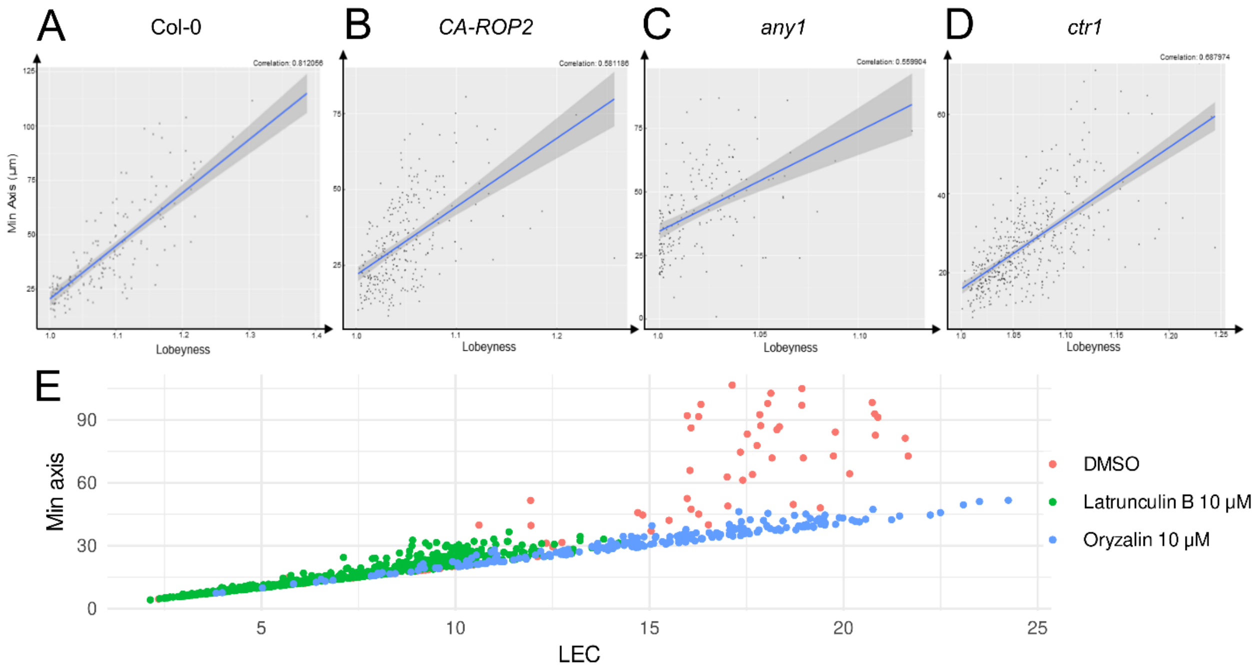

Appendix Fig. S4. Correlations between the min-axis and lobeyness in various mutants and with LEC in drug-treated plants. (**A**) Puzzle cells in the wild type exhibit a pronounced correlation between min-axis and lobeyness (Corr = 0.81). (**B-D**) This correlation is notably diminished in mutants: *CA-ROP2* (Corr = 0.58) (**B**), *any1* (Corr = 0.56) (**C**), and *ctr1* (Corr = 0.69) (**D**). (**E**) The scatter plot reveals the relationship between min-axis and LEC in pavement cells of drug-treated plants: DMSO control, latrunculin B 10 µM, and oryzalin 10 µM. The nearly linear trend in drug-treated plants suggests a diminished or absent capacity for these plants to form lobes.

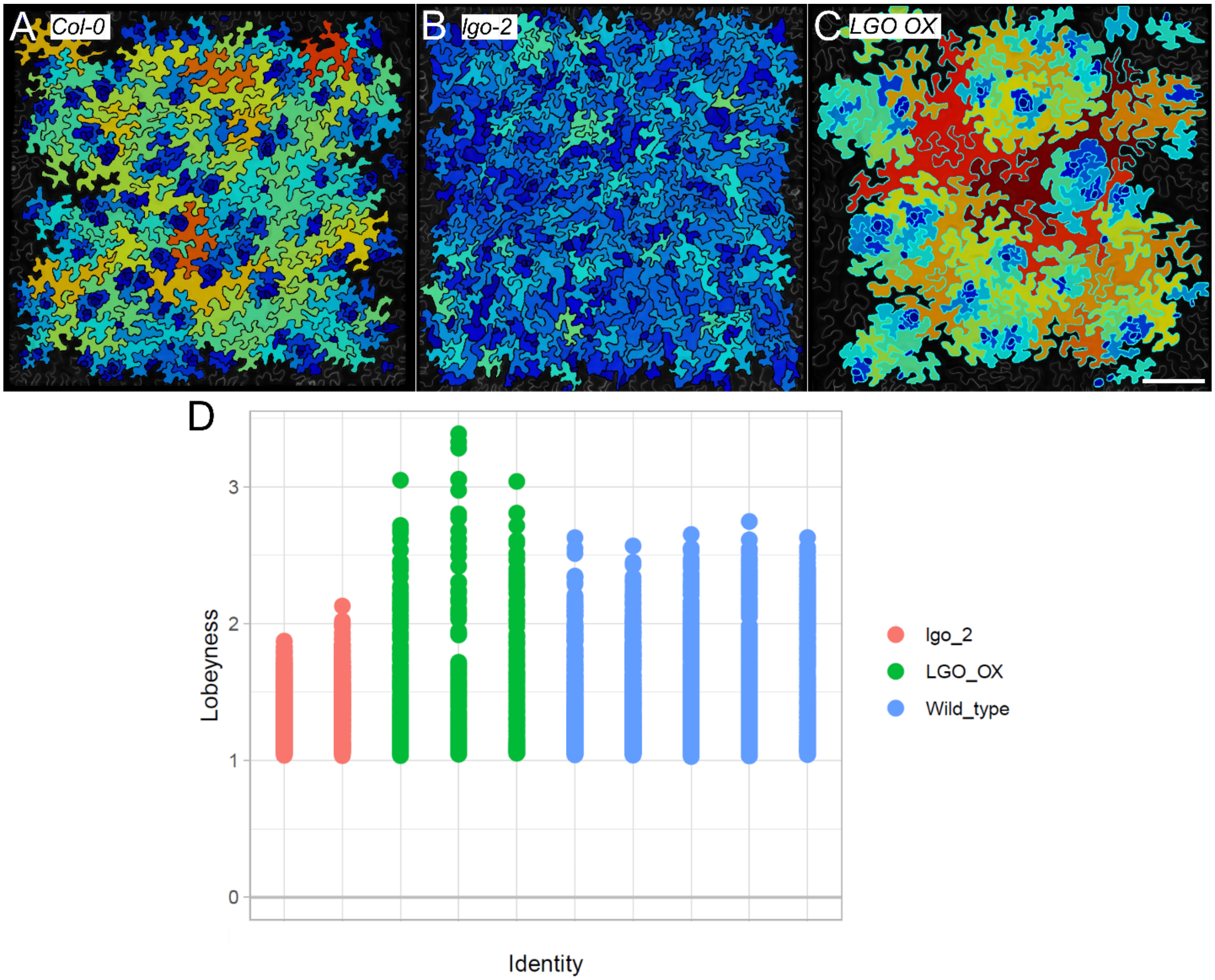

Appendix Fig. S5. Lobeyness of pavement cells is negatively correlated with cell division activity. (**A-C**) Heatmaps represent the lobeyness of pavement cells on the abaxial side of *Arabidopsis* leaves in the wild type (**A**), *lgo-2* mutant (**B**) and *LGO-OX* (**C**) at 25 days post germination. (**D**) Quantification of lobeyness in the wild type, *lgo-2* mutant, and *LGO-OX*. Reduced lobeyness is observed in the *lgo-2* mutant compared to wild type, while increased lobeyness is seen in the *LGO-OX*, indicating a negative correlation between pavement cell lobeyness and cell division activity regulated by *LGO*. Scale bar, 200 µm.

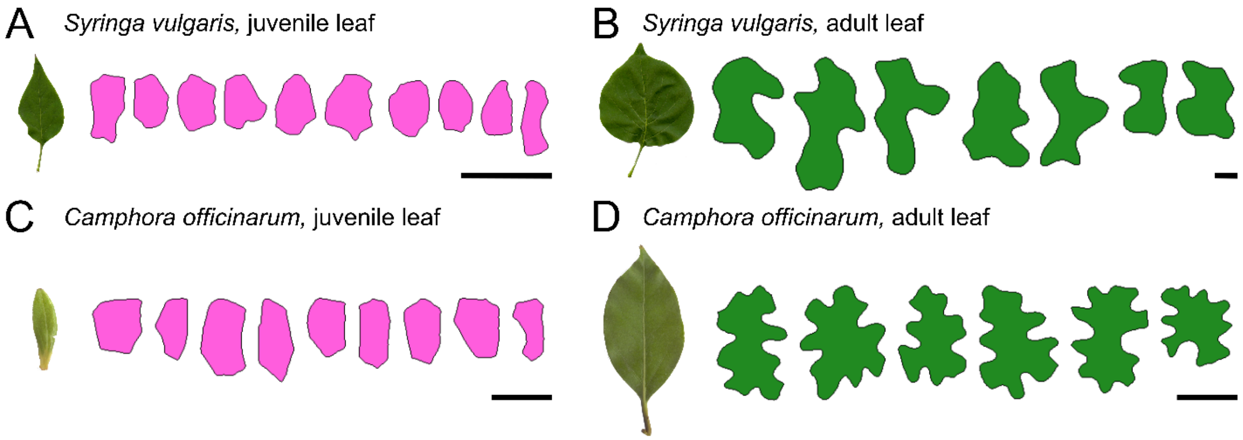

Appendix Fig. S6. Developmental variation in leaf pavement cell shapes. (**A-D**) Juvenile leaf and corresponding cell outlines (**A**) compared to adult leaf with more lobed cells (**B**) in lilac (*Syringa vulgaris*). Juvenile leaf and corresponding non-lobed cell outlines (**C**) compared to adult leaf with lobed cells (**D**) in camphor tree (*Camphora officinarum*) grown indoors. Different colors indicate cells sampled from: small leaves (pink), adult leaves (dark green). Scale bars for cell contours, 50 µm.

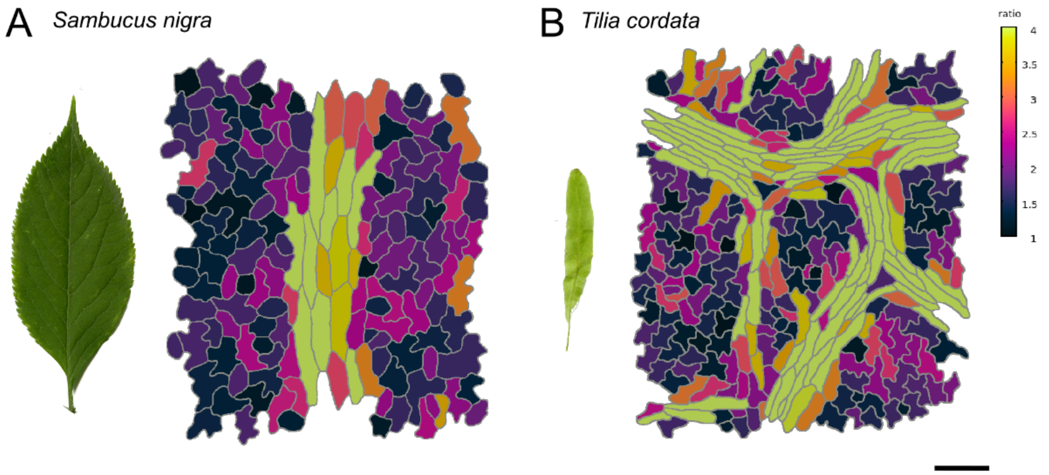

Appendix Fig. S7. Variation in epidermal pavement cell morphology on the organ surface. (**A**) Elderberry (*Sambucus nigra*) leaf and the corresponding pavement cell contours on its abaxial side. (**B**) Linden (*Tilia cordata*) bract and the corresponding pavement cell contours on its surface facing the flower. Cells are color-coded by aspect ratio heatmap. Scale bars for cell contours, 100 μm.

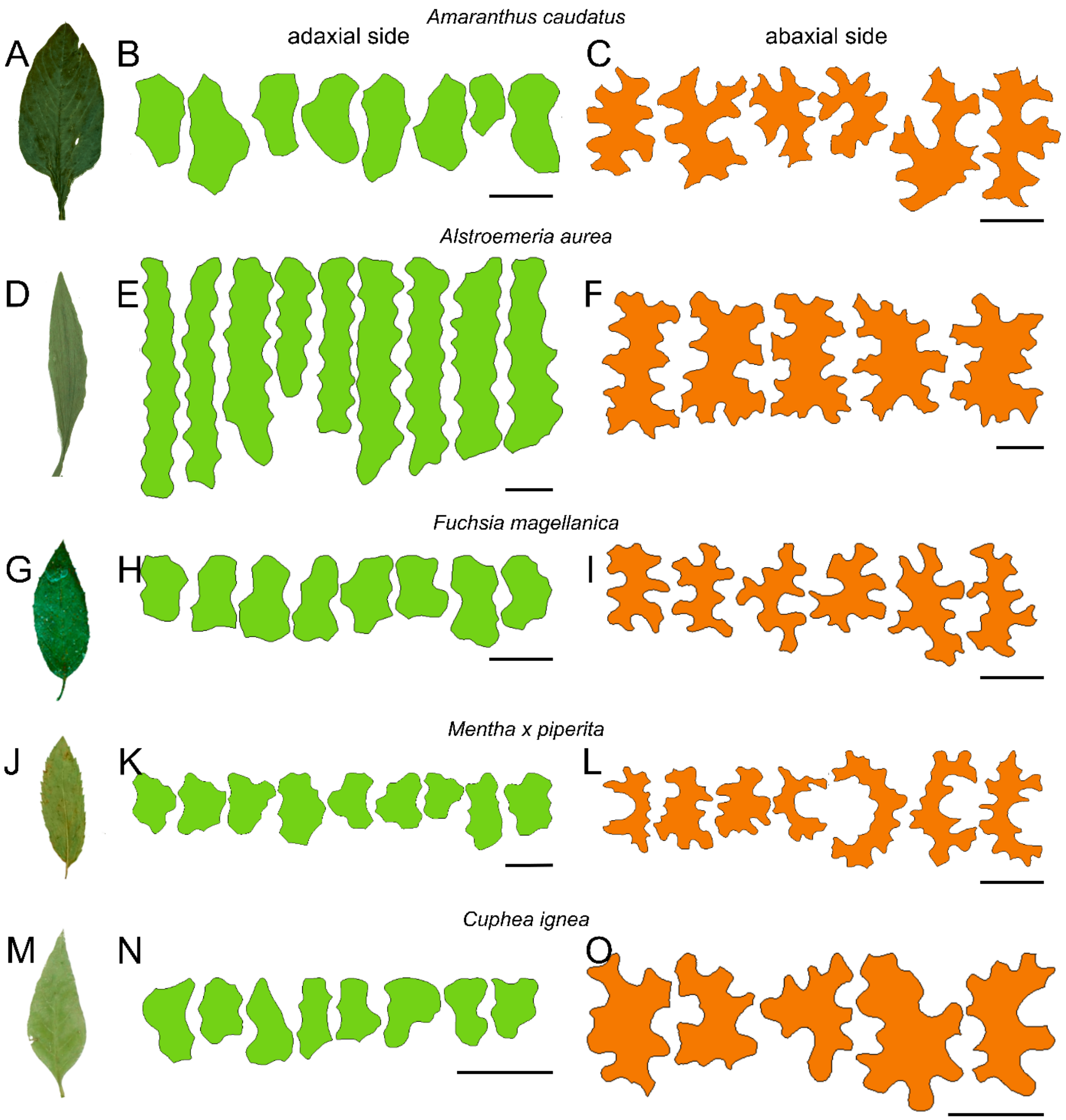

Appendix Fig. S8. The lobeyness of pavement cells varies between adaxial and abaxial leaf surfaces. (**A-O**) Leaf images (**A, D, G, J, M**) and corresponding pavement cell outlines representing the 95th percentile of lobeyness on the adaxial (cells colored in green) (**B, E, H, K, N**) and abaxial (cells colored in orange) (**C, F, I, L, O**) leaf surfaces. Each row represents a different species: love-lies-bleeding (*Amaranthus caudatus*) (**A-C**), Peruvian lily (*Alstroemeria aurea*) (**D-F**), fuchsia (*Fuchsia magellanica*) (**G-I**), peppermint (*Mentha* × *piperita*) (**J-L**), and cigar flower (*Cuphea ignea*) (**M-O**). Scale bar for cell contours, 150 µm.

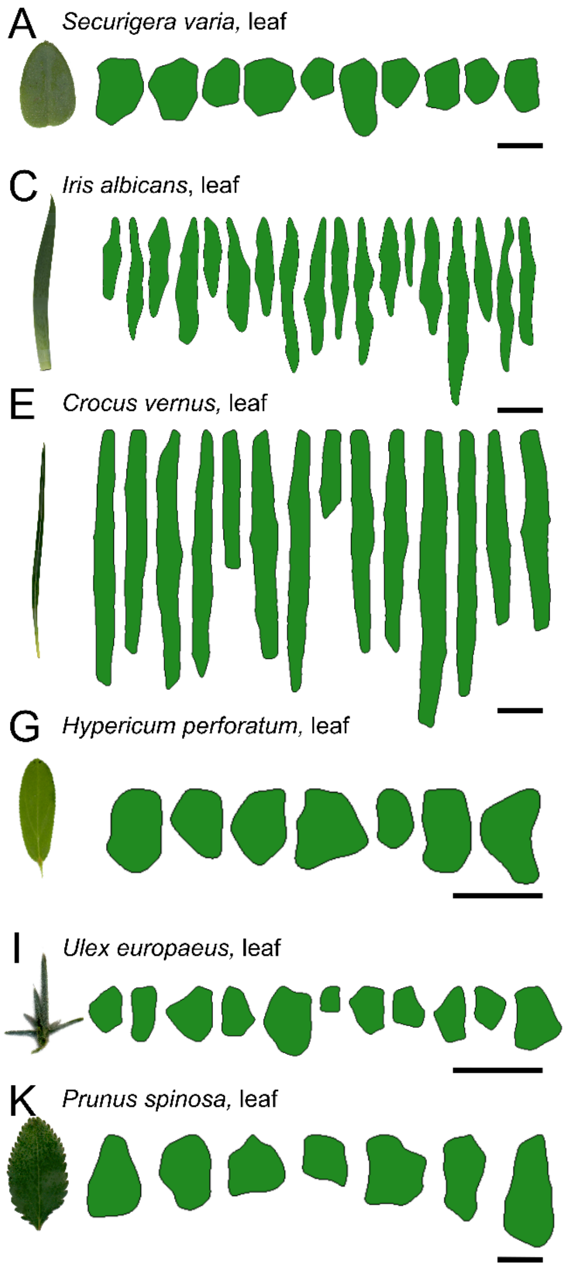

Appendix Fig. S9. Organ-specific differences in pavement cell shape between leaves and floral structures. (**A-L**) Leaf and corresponding non-lobed cell outlines (**A**, **C**, **E**, **G**, **I**, **K**) compared to lobed pavement cells of petals in crownvetch (*Securigera varia*) (**B**), cemetery iris (*Iris albicans*) (**D**), spring crocus (*Crocus vernus*) (**F**), and sepals in St John's wort (*Hypericum perforatum*) (**H**), gorse (*Ulex europaeus*) (**J**) and blackthorn (*Prunus spinosa*) (**L**). Different colors indicate cells sampled from: adult leaves (dark green); petal and sepal contours are colored to match the organ appearance when possible. Scale bars for cell contours, 50 µm.

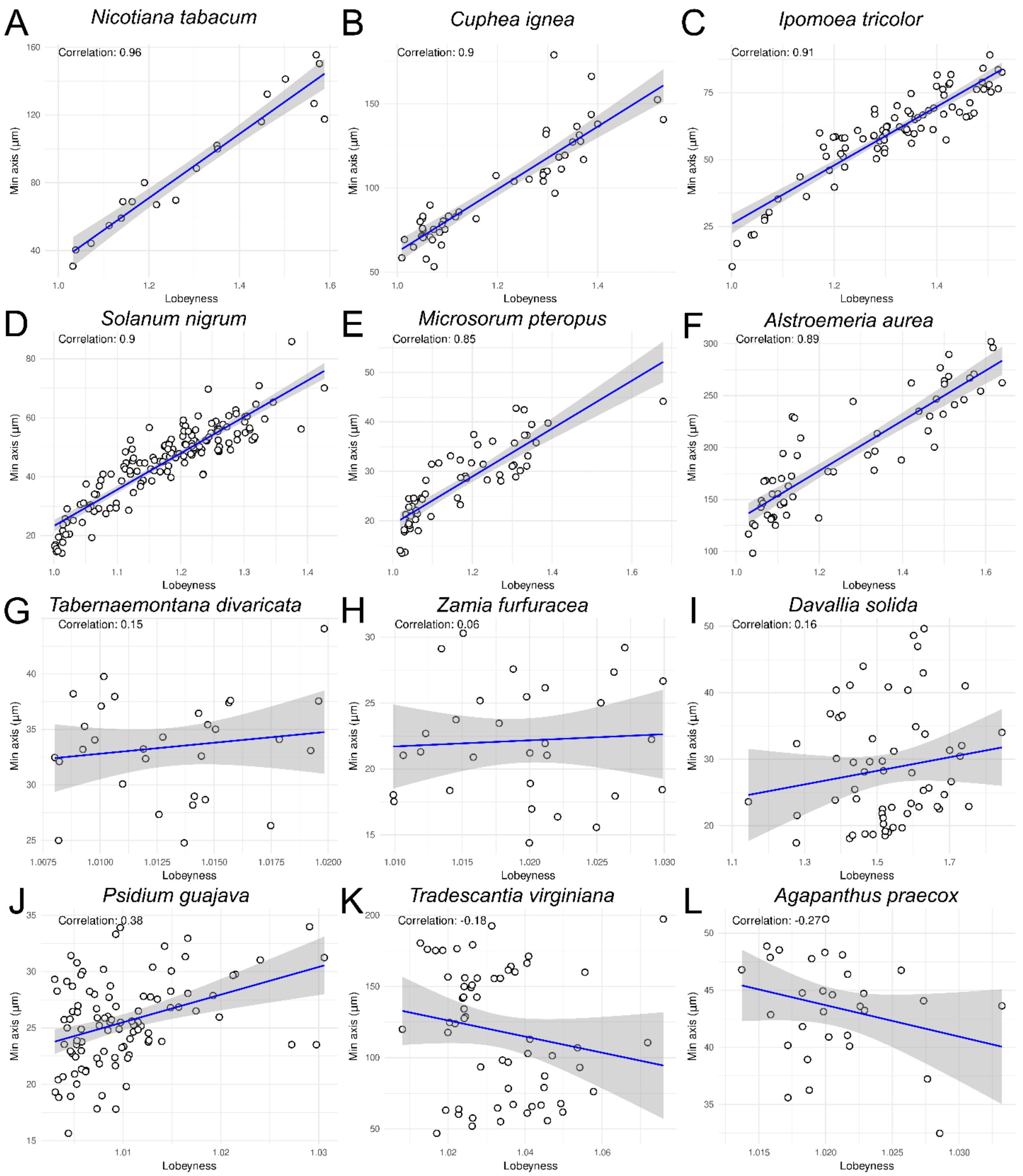

Appendix Fig. S10. Correlation patterns between min-axis and lobeyness in pavement cells within individual species. Each panel shows a scatter plot illustrating how the min-axis (the shortest cell dimension, y-axis) relates to lobeyness (x-axis) for a given species, revealing a spectrum of correlation strengths. (**A–F**) Examples of strong positive correlation in tobacco (*Nicotiana tabacum*) (**A**), cigar plant (*Cuphea ignea*) (**B**), morning glory (*Ipomoea tricolor*) (**C**), black

nightshade (*Solanum nigrum*) (**D**), Java fern (*Microsorum pteropus*) (**E**), and Peruvian lily (*Alstroemeria aurea*) (**F**). (**G–L**) Weak or negative correlation in pinwheel flower (*Tabernaemontana divaricata*) (**G**), cardboard palm (*Zamia furfuracea*) (**H**), hare's foot fern (*Davallia solida*) (**I**), yellow guava (*Psidium guajava*) (**J**), Virginia spiderwort (*Tradescantia virginiana*) (**K**), and agapanthus (*Agapanthus praecox*) (**L**).

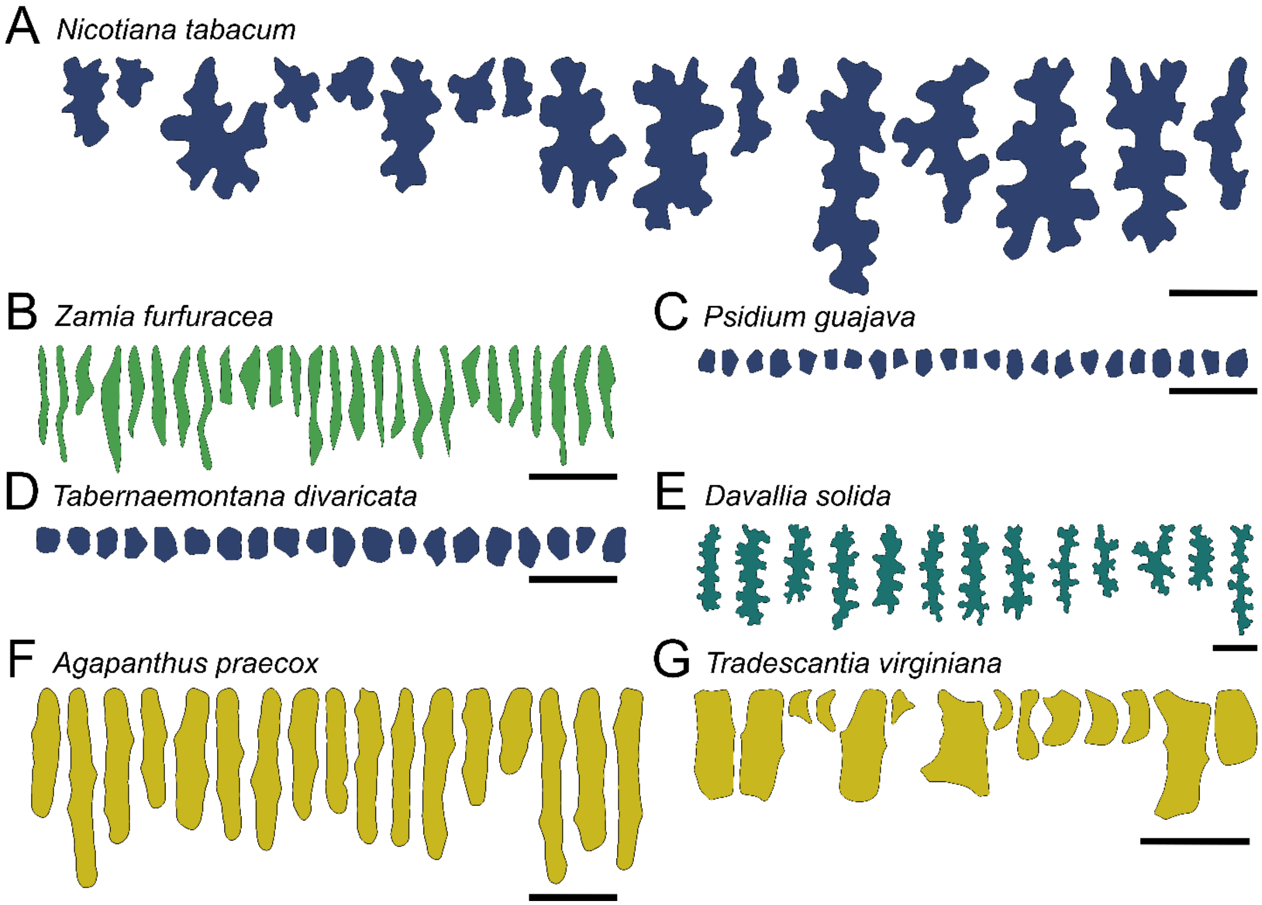

Appendix Fig. S11. Visual examples of pavement cell contours demonstrating high, low, and negative correlations between lobeyness and min-axis. (**A-G**) Cell contours of leaves color-coded by clade. (**A**) Pavement cells of tobacco (*Nicotiana tabacum*) that have a high correlation between min-axis and lobeyness (Corr = 0.97). (**B**) The cardboard palm cells (*Zamia furfuracea*) have a low correlation that is typical for long and thin cells (Corr = 0.24). (**C**, **D**) Epidermal cells in yellow guava (*Psidium guajava*) and pinwheel flower (*Tabernaemontana divaricata*) maintain small cells of a uniform size (Corr = 0.38 and Corr = 0.13, respectively). (**E**) Highly lobed cells in the hare's foot fern (*Davallia solida*) display little variation in lobeyness, which influences the correlation (Corr = 0.16). (**F**) In agapanthus (*Agapanthus praecox*), bumps next to junctions of neighboring cells affect thinner cells more than thicker ones, resulting in negative correlations (Corr = -0.21). (**G**) Negative correlations are also typical for pavement cells in the Virginia spiderwort (*Tradescantia virginiana*) with the much smaller stomatal lineage cells displaying irregular concave shapes (Corr = -0.2). Scale bars, 250 µm.

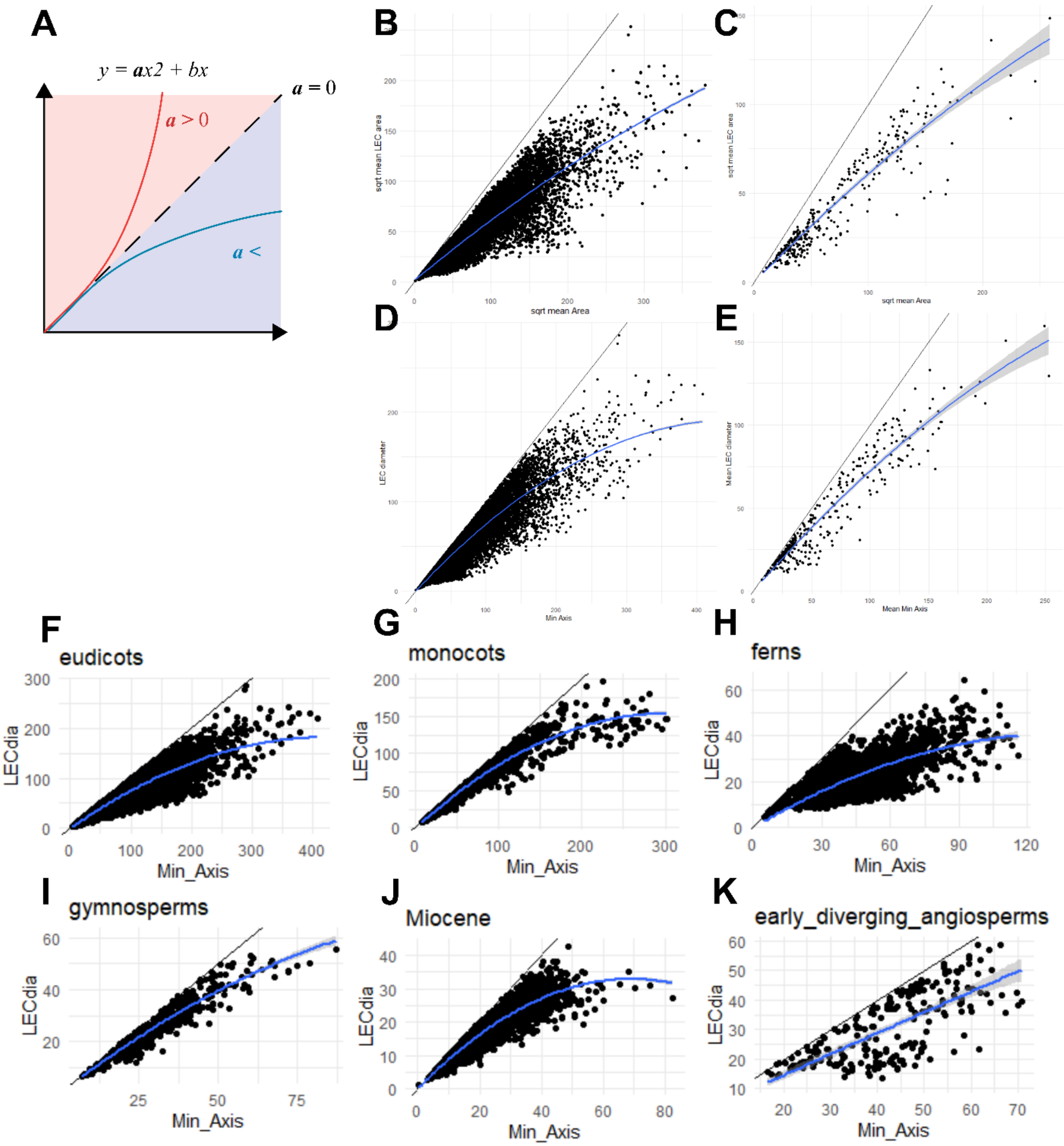

Appendix Fig. S12. The analysis of LEC across different species suggests a correlation between cell size and lobeyness. The black line in each plot indicates the theoretical limit where LEC area equals cell area (**B, C**), or LEC diameter equals min-axis length (**D-K**). The blue line indicates the best-fit quadratic polynomial passing through the origin, and the grey shaded region indicates the 95% confidence interval. (**A**) The sign of the coefficient on the quadratic term indicates whether LEC tends to increase more quickly ($\alpha > 0$) or slowly ($\alpha < 0$) as cell size increases. (**B-C**) LEC area vs. cell area for all cells (**B**) and species means (**C**). (**D-E**) LEC diameter vs. min-axis length for all cells (**D**) and species means (**E**). (**F-K**) LEC diameter vs. min-axis

length for all cells in the indicated clade. Negative α values were observed in 87% of the species included in this analysis (286/327 species, exact binomial test, $p = 2.32e-46$), with significant enrichment also observed in eudicots (172/197, $p = 8.45e-19$); monocots (38/49, $p = 1.42e-4$); ferns (40/41, $p = 3.82e-11$); gymnosperms (16/19, $p = 4.43e-3$); Miocene species (13/13, $p = 2.44e-4$); and early diverging angiosperms (7/7, $p = 0.0156$).

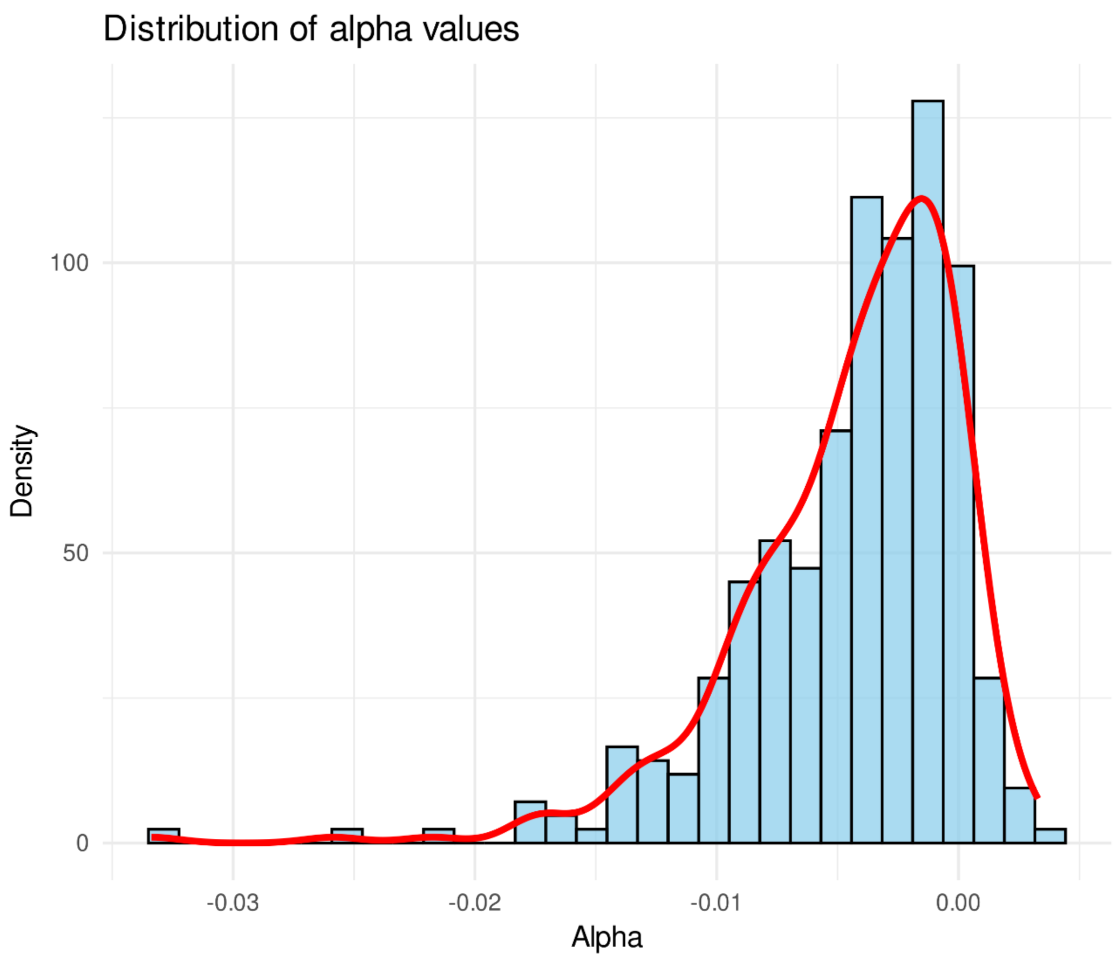

Appendix Fig. S13. Distribution of α values across species. The histogram shows the distribution of α values (bins = 30) computed from the quadratic fit of LEC versus min-axis. The red density curve represents a smoothed distribution. Most α values are negative, supporting the conclusion that LEC increases at a slower rate as the min-axis becomes larger. An exact sign test was performed to assess the probability of observing 286/327 negative coefficients by chance, confirming that this pattern is highly unlikely to occur randomly.
